# Primary Epstein-Barr Virus Infection in an Adolescent Female Complicated by Acute Acalculous Cholecystitis

**DOI:** 10.7759/cureus.5044

**Published:** 2019-06-30

**Authors:** Callan Young, Richard Lampe

**Affiliations:** 1 Pediatrics, Texas A&M College of Medicine, Dallas, USA; 2 Pediatrics, Texas Tech University Health Sciences Center, Lubbock, USA

**Keywords:** pediatrics, epstein-barr, acute acalculous cholecystitis, adolescent, epstein-barr virus (ebv), conservative management, infectious mononucleosis

## Abstract

Epstein-Barr Virus (EBV) is estimated to infect greater than 98% of adults worldwide and is one of the most common human viruses. EBV infection can lead to acute infectious mononucleosis characterized by fever, fatigue, malaise, sore throat, and lymphadenopathy. Elevated liver function tests (LFTs) and hepatosplenomegaly may also be present. This infection usually lasts over a period of weeks or months and is self-limited. Infected individuals, however, may suffer rare complications. Acute acalculous cholecystitis (AAC) is an atypical complication of infectious mononucleosis. The case of a young healthy adolescent female with primary EBV infection complicated by AAC is reported. Providers should be aware that many pediatric and adult cases of AAC due to EBV resolve with conservative treatment. Surgical intervention has not been described as necessary or indicated in the context of AAC caused by EBV and should only be considered in severe cases that are not responding to conservative therapy.

## Introduction

Epstein-Barr Virus (EBV) is estimated to infect greater than 98% of adults worldwide and is one of the most common human viruses [1]. Infection mainly occurs in early childhood and is predominantly asymptomatic. EBV infection can lead to acute infectious mononucleosis characterized by fever, fatigue, malaise, sore throat, and lymphadenopathy. Elevated liver function tests (LFTs) and hepatosplenomegaly may also be present. This infection usually lasts over a period of weeks or months and is self-limited. Infected individuals, however, may suffer rare respiratory, hematologic, hepatic, and/or psychological complications [1]. Acute acalculous cholecystitis (AAC) is an atypical complication of infectious mononucleosis. In this report, we present a case of primary EBV infection complicated by acute acalculous cholecystitis in an adolescent female.

## Case presentation

In August 2018, a previously healthy 14-year-old Caucasian female, body mass index (BMI) 22/Tanner Stage 5, presented to a regional hospital in Southern US with a three-day history of severe epigastric and right upper quadrant (RUQ) abdominal pain. She described the pain as sharp, almost constant, and worse with movement. She had been experiencing fatigue, nausea, vomiting, and anorexia for the past two to three weeks, which was treated with ondansetron. Past medical history was significant for Attention Deficit Hyperactivity Disorder (ADHD), for which she recently started lisdexamfetamine (Vyvanse) one month prior. Physical exam was significant for RUQ and epigastric tenderness without rebound or guarding. The remaining physical exam findings were unremarkable; she did not present with fever, cervical lymphadenopathy, or hepatosplenomegaly. Vital signs were stable. Her temperature was 37.6°C, heart rate was 70 beats per minute, respiratory rate was 20 breaths per minute, and blood pressure was 128/77 mmHg. Laboratory investigations revealed a white blood cell (WBC) count of 5.4 K/µL (within normal ranges) with elevated lymphocytes (45.2%) and monocytes (8.3%). The remainder of her complete blood count (CBC) and complete metabolic panel (CMP) were within normal ranges - aspartate aminotransferase (AST) 22 U/L, alanine aminotransferase (ALT) 13 U/L, and total bilirubin 0.2 mg/dL.

**Figure 1:**
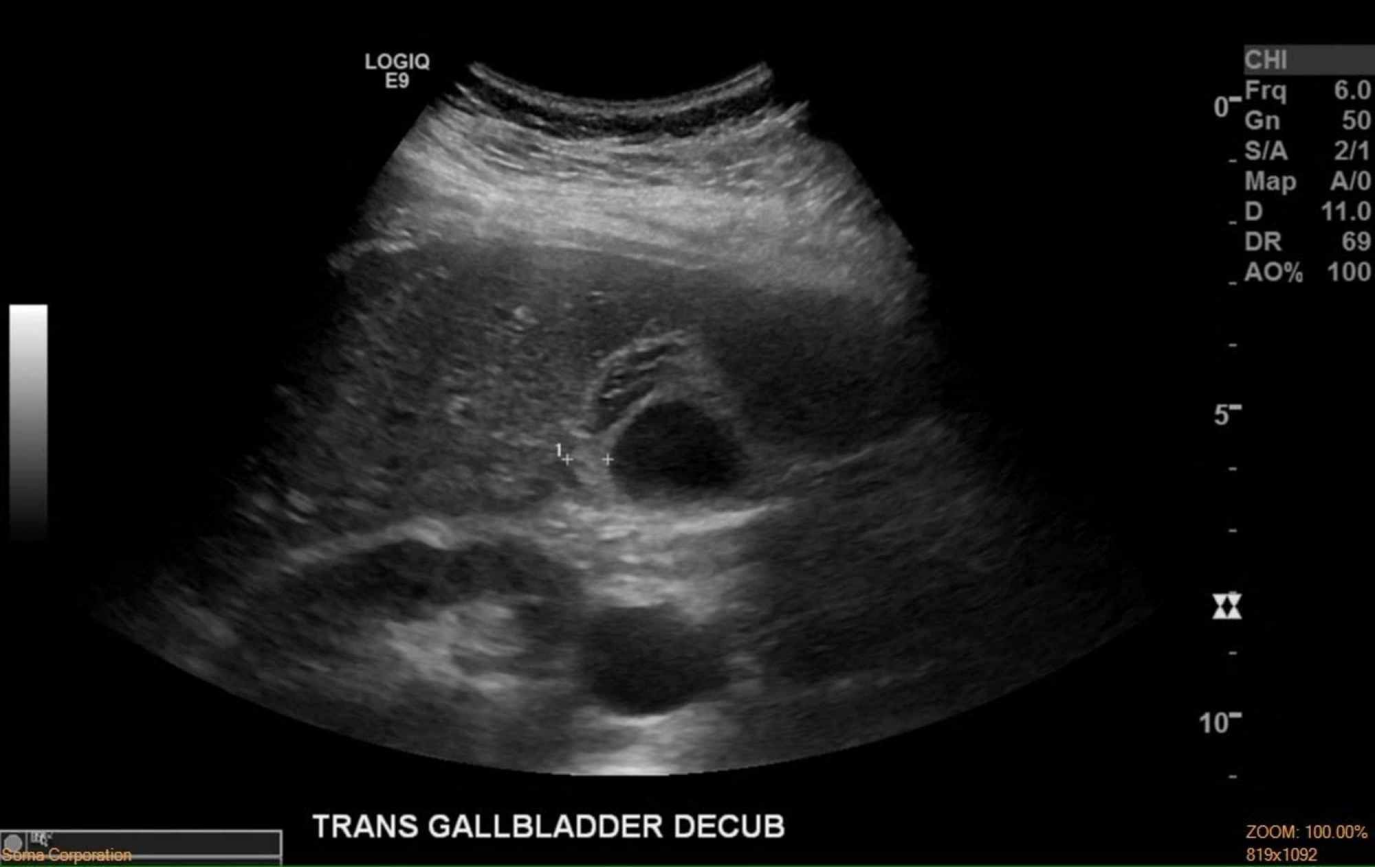
Gallbladder US showing 1.0 cm (10 mm) gallbladder wall thickening without the presence of gallstones or sludge cm = centimeters; US = ultrasound

Based on her presentation, computed tomography (CT) abdomen/pelvis with contrast was performed, as appendicitis was originally suspected. The CT scan was positive for only gallbladder wall thickening. Ultrasound of the gallbladder (Figure 1) was then performed, revealing a thickened gallbladder wall of 1.0 cm (10 mm) with associated pericholecystic fluid and elicitation of sonographic Murphy’s sign. The absence of gallstones and biliary sludge was noted. Nuclear medicine hepatobiliary scan with cholecystokinin (CCK) was performed and showed prompt clearance of the radiotracer from the gallbladder to the small bowel, indicating the absence of biliary obstruction. The patient experienced mild RUQ pain and nausea after the administration of CCK. The gallbladder motility study was abnormal with a gallbladder ejection fraction (GBEF) of only 24%. The normal GBEF fraction is greater than 35%. Acalculous cholecystitis was then diagnosed and a surgical specialist was consulted. She was placed on empiric antibiotics (Piperacillin-Tazobactam 4.5 g intravenous (IV) every six hours) and an infectious disease specialist was consulted. Surgery was deferred pending investigation of etiology and further diagnostic workup was performed.

Viral etiology was suspected given the patient's report of recent fatigue and sick contact exposure. Adenovirus, human metapneumovirus, parainfluenza, rhinovirus deoxyribonucleic acid (DNA) panel polymerase chain reaction (PCR) was negative. Blood culture was negative. The hepatitis panel was negative and lipase levels were unremarkable. EBV viral serology panel resulted in positive EBV viral capsid antigen (VCA) immunoglobulin M (IgM) and nuclear antigen immunoglobulin G (IgG) titers, indicating acute EBV infection.

After an extensive multi-provider discussion, it was decided to treat the condition conservatively with ibuprofen and acetaminophen for pain and ondansetron for nausea as needed. Empiric antibiotics were discontinued after 48 hours following the positive EBV viral serology panel and negative blood culture. The patient’s diet was advanced with full tolerance reached. Following significant improvement, the patient was discharged from the hospital two days later with close follow-up. Within two weeks following discharge, the patient was back to baseline activity with no residual abdominal pain or nausea. No follow-up imaging was performed due to full clinical improvement.

## Discussion

Acute acalculous cholecystitis (AAC) is an inflammatory disease of the gallbladder with a multifactorial pathogenesis. AAC accounts for only 10% of all cases of acute cholecystitis in adults but 50%-70% of cases of acute cholecystitis in children [2]. Diagnosis is based on a variety of symptoms including fever, abdominal pain, leukocytosis, and/or elevated liver function tests. Radiologic features include gallbladder wall thickening greater than 3 mm, gallbladder distention, pericholecystic fluid/sludge, and/or sonographic Murphy’s sign [3]. Of the above criteria, a combination of two or more is diagnostic in the clinical setting. AAC caused by etiologies other than viral agents are most commonly treated with antibiotics and/or cholecystectomy.

Acute acalculous cholecystitis is commonly caused by viral infectious agents in children, and rarely in adults. Cases have been reported of Epstein-Barr Virus, cytomegalovirus, hepatitis A virus, Dengue virus and, more recently, Zika virus leading to AAC [4-5]. In 2015, a literature review conducted by Agergaard and Larsen [6] found that 27 cases had been reported on AAC secondary to EBV infection. Twenty-six of the patients were females and one was male. Twenty-two of the patients reported gastrointestinal symptoms. Thickened gallbladder wall was the most consistent finding in all cases, ranging from 4.2 to 15 mm thickness. In 2016, a literature review performed by Kottanattu et al. [7] identified 37 cases of AAC caused by EBV. Thirty-two of the patients were female and five of the patients were male. These cases recovered conservatively without surgery or corticosteroids [7]. Upon a literature review, at least 12 cases of AAC caused by EBV have been reported since the year 2016 [4,8-18]. Eight patients were females and four patients were males. Three patients were children and adolescents: a two-year-old male, a 14-year-old female, and a 16-year-old female [13-14,17]. The aforementioned articles may imply a female gender predilection of AAC caused by EBV. However, more extensive research is required to properly establish this predilection.

Most cases of AAC caused by EBV in adults, adolescents, and children resolve without the need for surgical intervention. In 2009, Chalupa et al. [19] discussed the case of a 22-year-old with AAC caused by EBV with suspected perforation of the gallbladder. Surgical intervention was not performed, even though perforation was suspected, and she recovered with conservative management. In 2018, Rezkallah et al. [8] presented the case of a 25-year-old woman with AAC caused by EBV that required a laparoscopic cholecystectomy due to intolerable abdominal pain. The patient recovered fully following surgical intervention. Antibiotics are not considered necessary in AAC caused by EBV, due to its viral etiology. However, antibiotics may be considered if gallbladder perforation is suspected and secondary bacterial complications are imminent [19]. Similarly, antiviral medications show no evidence of benefit and are not commonly utilized for uncomplicated cases [1]. In children and adolescents, the current most-common therapeutic management of AAC is conservative treatment. Hospital admission should be considered to monitor clinical improvement and prevent complications. Providers should be aware that many pediatric and adult cases of AAC due to EBV resolve with conservative treatment and without surgical intervention [2]. The literature surrounding the surgical treatment of AAC caused by EBV infection is sparse. However, in 2018, Graber et al. [9] discussed the case of a 14-year-old female who underwent laparoscopic cholecystectomy for AAC that was later confirmed to be caused by EBV. She died approximately two hours after surgery due to unexplained postoperative complications.

## Conclusions

In conclusion, the case of a young healthy adolescent female with primary EBV infection complicated by AAC is reported. Our patient recovered fully with conservative treatment and without the need for surgical intervention. Surgical intervention has not been described as necessary or indicated in the context of AAC caused by EBV and should only be considered in severe cases that are not responding to conservative therapy.

## Appendices

Key: U/L = units/liter, K/µL = 1000/microliter, mg/dL = milligrams/deciliter
